# Social Network and Participation in Elderly Primary Care Patients in Germany and Associations with Depressive Symptoms—A Cross-Sectional Analysis from the AgeWell.de Study

**DOI:** 10.3390/jcm11195940

**Published:** 2022-10-08

**Authors:** Flora Wendel, Alexander Bauer, Iris Blotenberg, Christian Brettschneider, Maresa Buchholz, David Czock, Juliane Döhring, Catharina Escales, Thomas Frese, Wolfgang Hoffmann, Hanna Kaduszkiewicz, Hans-Helmut König, Margrit Löbner, Melanie Luppa, Rosemarie Schwenker, Jochen René Thyrian, Marina Weißenborn, Birgitt Wiese, Isabel Zöllinger, Steffi G. Riedel-Heller, Jochen Gensichen

**Affiliations:** 1 Institute of General Practice and Family Medicine, University Hospital of LMU Munich, 80336 Munich, Germany; 2 Institute of General Practice and Family Medicine, Martin-Luther-University Halle-Wittenberg, 06112 Halle (Saale), Germany; 3 German Center for Neurodegenerative Diseases (DZNE), Site Rostock/Greifswald, 17489 Greifswald, Germany; 4 Department of Health Economics and Health Services Research, University Medical Center Hamburg-Eppendorf, 20246 Hamburg, Germany; 5 Department of Clinical Pharmacology and Pharmacoepidemiology, University Hospital Heidelberg, 69120 Heidelberg, Germany; 6 Institute of General Practice, University of Kiel, 24105 Kiel, Germany; 7 Institute for Community Medicine, University Medicine Greifswald, 17487 Greifswald, Germany; 8 Institute of Social Medicine, Occupational Health and Public Health (ISAP), Medical Faculty, University of Leipzig, 04103 Leipzig, Germany; 9 MHH Information Technology, Medizinische Hochschule Hannover, 30625 Hannover, Germany

**Keywords:** aged, depression, social isolation, social networking, social participation

## Abstract

This study aims to describe social network and social participation and to assess associations with depressive symptoms in older persons with increased risk for dementia in Germany. We conducted a cross-sectional observational study in primary care patients (aged 60–77) as part of a multicenter cluster-randomized controlled trial (AgeWell.de). We present descriptive and multivariate analyses for social networks (Lubben Social Network Scale and subscales) and social participation (item list of social activities) and analyze associations of these variables with depressive symptoms (Geriatric Depression Scale). Of 1030 included patients, 17.2% were at risk for social isolation (Lubben Social Network Scale < 12). Looking at the subscales, a reduced non-family network was found almost twice as often as a reduced family network. Patients with depressive symptoms had significantly smaller social networks than patients without depression (*p* < 0.001). They rather engaged in social activities of low involvement level or no weekly social activity at all (*p* < 0.001). The study shows associations of depressive symptoms with a decreased social network and less social participation in elderly participants. Sufficient non-family contacts and weekly social activities seem to play an important role in mental health and should be encouraged in elderly primary care patients.

## 1. Introduction

Late life depression is an increasing public health concern and is estimated to be present in around 5.7% of the elderly population worldwide [1,2,3]. Depression is often undetected, especially in this patient group. Reasons comprise, for instance, unspecific or atypical presentation of symptoms as well as patient-reported discomforts mistakenly considered as merely part of the ageing process [4]. Consequently, depression is often not treated appropriately [5,6,7]. Depressive symptoms are associated with decreased cognitive, physical, and social functioning, with consequent implications for general wellbeing, multimorbid conditions and economic burden [8,9].

Social participation, as an important and also modifiable determinant of healthy aging, has received increasing attention in research and policy [10,11]. In Europe, only 20–30% of people aged 65–74 reported daily contact with family or relatives. Involvement in cultural or sporting events declined notably among adults aged 50–75 and above (61.6% vs. 35.6%) [12]. Social networks and belonging have been shown to be related to life satisfaction in older adults and to further impact physical and mental health outcomes [13,14,15,16,17,18,19].

Empowering individuals, families, and communities in increased social participation and addressing the broader determinants of health have been explicitly mentioned among the core components of Primary Health Care (PHC) [20]. Providing long-term care for patients and serving entire families, households and unions, primary care providers are in a crucial position to recognize depressive symptoms or signs of social isolation and to help patients with their medical and non-medical needs. The integration of mental health services into primary care is a recommended approach that has been implemented in many countries [21]. Apart from this, initiatives such as social prescribing seek to address the non-medical needs of patients by social interventions through collaboration of so-called link workers with primary care practices [22].

In this research, we examine associations between social participation and social networks and mental health. As such, we analyze depressive symptoms and how they are related with the social network size, family and non-family network, and social activities, according to two different involvement levels.

Pierre Bourdieu [23] described a concept referred to as social capital, which reflects the individual’s social network, social participation, and social support. Social participation broadly describes a person’s involvement in social activities [24], for example, community-based activities or interpersonal interactions, which rely on active engagement, resource sharing, and individual satisfaction [25]. Social networks comprise aspects of the structure and function of social connections, for example, the number, frequency, and reciprocity of ties [26]. In contrast, social support is considered a subjectively reported aspect. It has been suggested to be a mediator of the influence of social participation and social networks on health [26].

The objective of the present study is to describe the social networks and social participation of elderly community-dwelling primary care patients and to assess the associations with depressive symptoms.

## 2. Materials and Methods

### 2.1. Study Design

We performed a cross-sectional observational analysis in elderly primary care patients [27,28]. Data were collected as part of the baseline assessment of the AgeWell.de study, a multicenter cluster-randomized controlled trial. Baseline assessment was conducted between June 2018 and November 2019 at the five study sites in Northern, Eastern, and Southern Germany.

### 2.2. Recruitment Procedure

Community-dwelling primary care patients were recruited by their general practitioners (GPs) if they were between 60 and 77 years of age. A major inclusion criteria for study participation was a Cardiovascular Risk Factors, Aging and Incidence of Dementia (CAIDE) score [29] of > = 9 as an indicator for an increased risk for cognitive decline. CAIDE items comprised age, education, sex, blood pressure, BMI, cholesterol, and physical activity. As in the current analysis we do not focus on cognitive decline, a CAIDE score > 9 was interpreted as a proxy for the multimorbidity and social determinants of health of elderly patients. Exclusion criteria comprised, among others, suspected or diagnosed dementia or a severe clinical depression and other medical conditions potentially affecting engagement in the intervention [28].

### 2.3. Measurements

The outcomes analyzed in the present work are part of an extensive baseline questionnaire including standardized cognitive tests. Items were patient-reported in a face-to-face interview with the study nurse at the patient’s home, while cognitive tests were assessed by the study nurse.

For the present analysis, we used scores of the validated Geriatric Depression Scale (GDS) [30,31] to assess depressive symptoms, and the Lubben Social Network Scale (LSNS-6) [32] and Montreal Cognitive Assessment (MoCA) [33] to measure cognitive performance. We describe characteristics of the study participants using sociodemographic variables such as age and sex, and socioeconomic variables such as education (CASMIN), marital status, and employment.

### 2.4. Measures of Social Network

The LSNS-6 aims to capture the number of contact persons, received support, and trust. It consists of six questions, three concerning family (kinship, relatives) and three concerning non-family (friends, acquaintances). Whereas a LSNS-6 total score of <12 indicates a risk for social isolation according to the definition of the scale, a cut-off of 6 was established for the family and non-family subscales [32].

### 2.5. Measures of Weekly Social Activity and Social Participation

We used a standardized questionnaire on social activity (self-constructed items). As such, we analyzed if patients participated in a social activity at least once a week and which specific social activities of a proposed selection they participated in. The self-constructed items were dichotomous (yes/no) and can be found in the Supplementary Materials (Table S1).

To assess the social activities in terms of their level of involvement with others and the goal of the activity, we used a framework developed by Levasseur et al. [34]. This framework divides activities into the following categories: “(1) doing an activity in preparation for connecting with others, (2) being with others, (3) interacting with others without doing a specific activity with them, (4) doing an activity with others, (5) helping others, and (6) contributing to society” [34]. The authors discuss these levels as a continuum that depicts social participation (level 3 to 6) and that can enable distinguishing different concepts with more active involvement, such as social engagement (levels 5 and 6). Therefore we took a pragmatic approach by categorizing the social activities stated by the patients into activities that aim to help others or contribute to society as “involvement level high” (Taxonomy levels 5 and 6, also called social engagement [34]), activities with social interaction with or without a common goal as “involvement level low” (Taxonomy levels 3 and 4) and patients that did not participate in such social activities regularly as having “no social involvement” (Taxonomy level 1 or 2, or no social activity).

The considered social activities included social hobbies like card playing, dancing, sports groups, and visits to restaurants, bars, cinemas, or theatres (involvement level low), engagement in a political organization, club, association, religious institution or other volunteer occupation (involvement level high) and had to be carried out on a regular basis. If patients participated in several activities, we only considered the activity of the highest taxonomy stage.

### 2.6. Trial Registration and Ethical Clearance

The AgeWell.de trial is registered in the German Clinical Trials Register (DRKS; trial identifier: DRKS00013555) and approved by the responsible ethics boards of all participating study sites.

### 2.7. Statistical Analyses

Characteristics of the study cohort were described by absolute and relative frequency, as well as mean, median and standard deviation. The GDS was calculated for all participants answering at least 13 of 15 questions, whereas the LSNS-6 was only determined if all six questions were answered. As missing data—missing completely or at random— was estimated to be low (<2%), analyses were conducted without imputation, with *n* = 1016 and *n* = 1020, respectively. Chi-square tests were applied to examine differences between participants with and without risk for social isolation.

In order to examine the relationship of multiple variables with LSNS-6 > = 12 (not at risk for social isolation) and to control for confounding, we performed a logistic regression. Variables in this analysis included sociodemographic variables, marital status, education, employment, MoCA, and GDS. Variables were included simultaneously and chosen prospectively based on clinical relevance.

To assess associations between LSNS-6 and GDS, we used Spearman´s Rho. Associations of weekly social activity and LSNS-6 subscale groups with depressive symptoms were calculated using Pearson´s chi-square. Lastly, a linear trend test was used to examine associations between social participation (involvement levels) and the (categorial) LSNS-6 with GDS.

A *p*-value of <0.05 was determined to indicate significance of differences. Analyses were conducted using IBM SPSS^®^ Statistics Version 27, and multivariate analyses using STATA Version 16.

## 3. Results

A total of 1030 participants of the AgeWell.de study were included in the present analysis. Missing data of GDS and LSNS were attributable to patients not able or not willing to answer (part of) the questions. The characteristics of patients are displayed in Table 1. The mean age was 68.95 years, with 52.1% females. The majority of individuals were married and living with the partner (62.8%) and were retired or unemployed (78.68%). The MoCA mean score was 24.33, indicating that a light cognitive impairment was present in the study sample.

### 3.1. Description of Social Network, Social Participation, and Depressive Symptoms in the Study Participants

Among the participants, 5.5% had symptoms of a light to moderate depression. Only 0.4% of patients had severe depression. In terms of the social network size, 17.2% of patients were considered at risk for social isolation. Limited social contacts were found regarding the non-family network more than twice as often (25.3% of patients) as regarding the family network (11.8% of patients). Social activities with a low involvement level were reported by almost half of the participants (47.4%), and activities with a high involvement level by 39.4%. However, a social activity with a frequency of at least once a week was only indicated by 57.4% of all participants.

### 3.2. Multivariate Analysis of Variables That Influence Social Networks

Multivariate analyses revealed the significant influence of sex on LSNS-6 and subscales, meaning that female participants were less likely to be at risk for social isolation. Divorced participants were significantly more likely to have a sufficient non-family network and do a weekly social activity; however, they were more likely to have an insufficient family network. An insufficient family network was also found for all other participants with the exception of the married participants living together. Compared to individuals with a low education level, participants with a middle education level engaged in social activities with a higher involvement grade.

Results of the multivariate analysis to control for age and sex and to examine the influence of several variables on the LSNS-6 and other social variables are displayed in Table 2. Controlling for the main sociodemographic variables in this regression analysis, the negative association of the GDS score with the LSNS-6 score and all analyzed social variables remained significant.

### 3.3. Associations of Depressive Symptoms with Social Networks, Weekly Social Activity, and Social Participation

Patients with depressive symptoms had significantly fewer social contacts than patients without depression (Pearson Chi square test, *p* < 0.001). Data are displayed in Table 3. The association of GDS score and LSNS-6 score was significantly negative (r = −0.218, *p* < 0.001). Depressive symptoms were found more frequently in patients with a restricted non-family network and a normal family network than in patients with a normal non-family network and a restricted family network (Pearson chi-square test *p* < 0.001). Patients with depression participated in social activities of low involvement level or had no regular social activity at all (Linear-by-Linear Association, *p* < 0.001).

## 4. Discussion

Our research shows that depressive symptoms are significantly associated with decreased social network size and less social participation in elderly patients. Apart from the LSNS-6, we looked at its subscales separately as well as the involvement level of social activities to provide a broader picture of social participation. The associations of the GDS on all the observed variables of social network, participation, and activity remained significant, though possible confounders were included in the multivariate analysis model. These findings align with previous research reporting a decreased social network as well as a lack of social participation (volunteering, attendance in social centers, religious services) associated with depression in older adults [35,36] and persistent associations even after controlling for major confounders [32,37].

The characteristics of the analyzed elderly GP patients at risk of dementia differ in some ways from other studies examining elderly populations in Germany. As such, our population was younger (median age 68.95 vs. 73.86 [38] and 74.4 [39]) and displayed more chronic conditions such as hypertension [39,40], a higher BMI [21,38,41], and less physical activity [41,42,43]. These differences possibly derive from the selection of the AgeWell.de study participants based on the CAIDE score. In addition, the relatively small amount of participants with severe depression in our study population can be explained by exclusion of persons already diagnosed with depression.

Depressive symptoms and social participation have been examined cross-sectionally in the German elderly population by other studies. The prevalence of depressive symptoms among the older German population has been reported to be between 6.7% [1] and 17.7% [2,44,45]. As in the present research we only included patients without clinical severe depression, our prevalence (GDS > 5) was found to be somewhat lower (5.9%). Generally, an increase in risk of depression with higher age among the elderly has been described [46].

To describe social participation or related terms like social network, support, engagement, and isolation, various concepts and measurement tools exist, impeding a direct comparison of findings [26,34]. The MultiCare study [47] reported the perceived social support in the cohort (Fragebogen zur sozialen Unterstützung, F-SozU-K14: Mean: 4.1, SD 0.69, 1: no support; 5: high support [44]), whereas the KORA-Age study [48] reported the University of California, Los Angeles (UCLA) Loneliness Scale (Mean: 17.15; Score range 12–48; higher scores suggesting more loneliness) and the Social Network Index (63.67% with low Social Network Index [2]). Data from Switzerland show 21.2% of participants being relatively inactive, 50.8% moderately active, and 27.9% highly active in a latent class analysis of social activities [11].

In the present study, patients with light to moderate depression participated in social activities of low involvement level and more often did not participate in a weekly social activity at all compared to patients without depression. However, in the literature, clear mechanisms or pathways are not fully understood, and findings often suggest bi-directional influences. For instance, Santini et al. [49] found in a longitudinal study that social disconnectedness predicted perceived isolation, which then predicted higher depressive symptoms. Herbolsheimer et al. [50] found social isolation associated with lower levels of out-of-home physical activity, which in turn was a predictor for depressive symptoms.

Further, we found an association of depressive symptoms, especially with a decreased non-family network. Patients with depressive symptoms were more likely to report a restricted non-family network, even if they had a normal family network. Our findings therefore suggest the importance of a social network outside the family circle and hypothesize that a normal family network cannot offset an insufficient non-family network in elderly patients. On the other hand, these findings might reflect the effects of social withdrawal, a symptom of depression.

Analyses comparing family and non-family networks in regard to their role in depression are scarce [32]. Holtfreter et al. [51] found that high-quality familial ties moderated the association of social participation and depressive symptoms. However, non-family ties were not considered in their analysis. Zhang et al. [52] described family support to be a mediator and friend support to be a moderator of the effects of marital status on depressive symptoms. Generally, family and non-family networks seem to play important roles in psychological needs while being suggestible to some extent. They assumably have different functions for elderly persons that are yet not fully understood [52]. Our findings thereby contribute to examining the role of non-family networks regarding depression and should be explored in further research.

Compared to previous analyses of German cohorts [2,44,45] of elderly patients, we examine new items of social participation (validated LSNS-6, subdomains of the LSNS-6, and social activities, taking into account the involvement level as well as the regularity of activities), thereby adding knowledge to the field of social participation, with its plurality of concepts and determinants.

### 4.1. Weaknesses

A limitation of the present study is the use of unvalidated tools to capture social participation. Further, we did not take into account the total number of different activities but focused on the regularity of certain activities (at least once a week) and only considered the activity with the highest involvement level according to an established taxonomy. The inaccuracy of details on certain social activities cannot be precluded, because we had to rely on patient-reported outcomes for most of the variables related to social participation. We analyzed social networks and social participation but did not assess social support as a separate outcome. This is partly reflected in the LSNS and further described to be a mediating variable between the other two outcomes [26].

Limitations in terms of the broader study design comprise a potential selection of participants through recruitment by GPs and the inclusion of only patients with an increased risk of cognitive decline (CAIDE score > = 9). Hence, the generalizability of results for the general public above 60 years might be limited. On the other hand, the multicentered study design and including participants from rural and urban areas at each study site suggests the generalizability of results within Germany for persons with similar characteristics to the study participants. Further, the rate of consenting GPs of all contacted GPs for study participation was poor, at 17.86%, probably because recruitment of patients was linked to the participation of GPs in the study. In studies that only require the provision of information by the GPs, response rates are usually higher, for example, 72.5% [53]. The dropout rate of patients in our study, however, was relatively low, at 8.67%, compared to other studies [48,53].

### 4.2. Implications for Clinical Practice

Depressive symptoms as well as social networks and participation are modifiable determinants of healthy aging that seem interconnected and therefore should be addressed equally. GPs and primary care teams must be aware of different presentations of depressive symptoms in elderly patients and ensure appropriate treatment, including non-pharmaceutical interventions and support adherence. Models of integrated care catering also to mental health and social needs, delivered by a multidisciplinary team, seem to be a promising approach. Medical history must include social participation, explicitly asking about social networks and regular social activities. Elderly patients should be empowered to sustain their social network and to engage in a social activity at least weekly, preferably with a high involvement grade.

### 4.3. Implications for Research

Further research is needed to explore social participation and social networks more deeply and to clarify mechanisms of influence on depressive symptoms and health. More longitudinal analyses are needed, examining potential mechanisms and mediators of influence, and controlling for possible confounders. Apart from this, future research should develop and use more standardized concepts and assessment batteries to capture social participation and networks in clinical trials and observational studies. The integration of qualitative methods could help to explore perspectives and needs of patients and care providers and to develop and improve tailored interventions.

## 5. Conclusions

A considerable share of the observed elderly primary care patients was at risk of social isolation and lacked regular social activities. Having an insufficient non-family network was observed about twice as often as an insufficient family network. Male participants were more likely to have a decreased social network generally, whereas unmarried or individuals living apart were more likely to have a sufficient non-family network. In the observed elderly patients, an insufficient social network was associated with more depressive symptoms. Patients with depressive symptoms showed smaller networks and engaged in social activities of low involvement level with others or had no regular weekly social activity at all compared to patients without depression.

## Figures and Tables

**Table 1:** Characteristics of included patients (*n* = 1030).

	Absolute (Relative) Frequency	LSNS-6 > = 12	LSNS-6 < 12	Chi-Square Tests
		*n* = 1020		
**Sex**				0.057
female	537 (52.1%)	453 (53.6%)	80 (45.7%)	
male	493 (47.9%)	392 (46,4%)	95 (54.3%)	
**Age** (Mean, SD)	68.95 (4.94)	68.85 (4.91)	69.42 (5.04)	0.162
**Education (CASMIN)**				0.054
Low	251 (24.4%)	201 (23.8%)	48 (27.4%)	
Middle	546 (53%)	442 (52.3%)	98 (56%)	
High	233 (22.6%)	202 (23.9%)	29 (16.6%)	
**Marital status**				0.003
Married, living together	647 (62.8%)	550 (65.1%)	89 (50.9%)	
Married, living apart	24 (2.3%)	20 (2.4%)	4 (2.3%)	
Partnership	18 (1.7%)	14 (1.7%)	4 (2.3%)	
Single	48 (4.7%)	32 (3.8%)	16 (9.1%)	
Divorced	129 (12.5%)	99 (11.7%)	29 (16.6%)	
Widowed	164 (15.9%)	130 (15.4%)	33 (18.9%)	
**Employment** *n* = 1018				0.006
Employed (full-/part-time/mini-job)	217 (21.32%)	645 (77%)	147 (86.5%)	
Retired/unemployed	801 (78.68%)	193 (23%)	23 (13.5%)	
**MoCA** (Mean, SD) *n* = 1026	24.33 (3.14)	24.5 (3.04)	23.51 (3.41)	0.000
**GDS** (Mean, SD) *n* = 1016	1.62 (2.03)	1.39 (1.79)	2.66 (2.64)	0.000
**GDS**: normal	956 (94.1%)	810 (96.8%)	142 (81.6%)	0.000
Light/moderate depressive symptoms	56 (5.5%)	25 (3%)	30 (17.2%)	
Severe depression	4 (0.4%)	2 (0.2%)	2 (1.1%)	
**Social activity** *n* = 1030				0.000
No involvement	136 (13.2%)	76 (9%)	52 (29.7%)	
Involvement level low	488 (47.4%)	401 (47.5%)	86 (49.1%)	
Involvement level high	406 (39.4%)	368 (43.6%)	37 (21.1%)	
**Weekly social activity** *n* = 1015	583 (57.4%)	531 (63.5%)	51 (29.3%)	0.000
**LSNS-6** (Mean, SD) *n* = 1020	17.13 (5.63)			
LSNS-6 > = 12	845 (82.8%)			
LSNS-6 < 12	175 (17.2%)			
LSNS family < 6	120 (11.8%)			
LSNS family > = 6	900 (88.2%)			
LSNS non-family < 6	258 (25.3%)			
LSNS non-family > = 6	762 (74.7%)			

GDS: Geriatric depression scale: 0–5: normal; 6–10: light to moderate depression; 11–15: severe depression. LSNS-6: Lubben Social Network Scale: 12–24: not at risk for social isolation; 0–11: at risk for social isolation. MoCA: Montreal Cognitive Assessment: 26–30: normal; 0–25: cognitive impairment.

**Table 2:** Multivariate analyses (logistic regression) OR (95% CI); *p*-value.

	LSNS-6 < 12 (at Risk for Isolation)	LSNS-6 Family < 6 (at Risk for Isolation)	LSNS-6 Non-Family < 6 (at Risk for Isolation)	A Social Activity at least 1/Week	Social Participation Involvement Grade (Higher)
**Age**	1.02 (0.98–1.06); 0.259	1.02 (0.97–1.07); 0.494	0.99 (0.96–1.03); 0.697	1.03 (1.00–1.06); 0.078	1.02 (0.99–1.05); 0.135
**Sex** female	0.63 (0.44–0.92); 0.016 *	0.46 (0.29–0.73); 0.001 *	0.70 (0.51–0.96); 0.026 *	1.31 (0.99–1.74); 0.061	0.95 (0.74–1.23); 0.720
**Marital status**					
Married, living apart	1.18 (0.38–3.71); 0.776	2.18 (0.06–7.89); 0.235	0.50 (0.16–1.54); 0.227	1.22 (0.50–2.93); 0.665	0.75 (0.34–1.64); 0.469
Partnership	1.47 (0.46–4.70); 0.516	4.99 (1.64–15.14); 0.005 *	0.46 (0.13–1.64); 0.229	1.48 (0.55–3.99); 0.441	0.96 (0.38–2.42); 0.928
Single	2.98 (1.48–6.02); 0.002 *	9.56 (4.57–20.01); 0.000 *	0.89 (0.43–1.85); 0.753	0.57 (0.29–1.11); 0.099	0.69 (0.39–1.23); 0.204
Divorced	1.59 (0.95–2.66); 0.079	4.02 (2.24–7.19); 0.000 *	0.54 (0.32–0.90); 0.019 *	1.66 (1.07–2.57); 0.025 *	1.34 (0.91–1.97); 0.140
Widowed	1.50 (0.91–2.45); 0.109	2.97 (1.64–5.36); 0.000 *	0.75 (0.47–1.17); 0.201	1.01 (0.68–1.51); 0.942	1.07 (0.75–1.53); 0.697
**Education (CASMIN)**					
middle	1.19 (0.78–1.81); 0.430	1.06 (0.64–1.77); 0.808	1.28 (0.89–1.86); 0.188	0.75 (0.54–1.05); 0.096	0.65 (0.48–0.88); 0.006 *
high	0.91 (0.51–1.60); 0.736	0.90 (0.46–1.77); 0.756	0.74 (0.45–1.22); 0.242	1.33 (0.87–2.03); 0.186	1.15 (0.79–1.67); 0.468
**Employment**	0.63 (0.37–1.06); 0.082	0.66 (0.36–1.23); 0.196	0.74 (0.48–1.12); 0.154	1.30 (0.91–1.87); 0.150	1.10 (0.80–1.51); 0.553
**GDS score**	1.25 (1.16–1.35); 0.000 *	1.16 (1.07–1.26); 0.001 *	1.24 (1.15–1.33); 0.000 *	0.81 (0.75–0.87); 0.000 *	0.82 (0.77–0.87); 0.000 *
**MoCA score**	0.95 (0.90–1.01); 0.099	0.98 (0.91–1.05); 0.523	0.95 (0.90–1.00); 0.043 *	1.11 (1.06–1.16); 0.000 *	1.07 (1.02–1.12); 0.002 *

* Significant at the 0.05 level.

**Table 3:** Associations of social network, activity, and participation with depressive symptoms.

	GDS: Normal	GDS: Light to Severe Depression
**Social participation***n* = 1016	Linear-by-linear Association: *p* < 0.001	
No involvement	112 (86.2%)	18 (13.8%)
Involvement level low	451 (93.6%)	31 (6.4%)
Involvement level high	393 (97.3%)	11 (2.7%)
**Weekly social activity***n* = 1008	Pearson´s Chi-Square-test: *p* < 0.001	
At least 1/week	561 (96.7%)	19 (3.3%)
No weekly social activity	388 (90.7%)	40 (9.3%)
**LSNS-6** *n* = 1011	Linear-by-linear Association: *p* < 0.001	
LSNS-6 > = 12	810 (86.9%)	27 (3.2%)
LSNS-6 < 12	142 (81.6%)	32 (18.3%)
**LSNS-6 subscales**	Pearson´s Chi-Square-test: *p* < 0.001	
Family > 6/non-family > 6	664 (97.6%)	664 (97.6%)
Family > 6/non-family < 6	190 (88.8%)	190 (88.8%)
Family < 6/non-family > 6	66 (90.4%)	66 (90.4%)
Family < 6/non-family < 6	32 (72.7%)	12 (27.2%)

## Data Availability

Data are available upon request.

## Supplementary Materials

The following supporting information can be downloaded at: https://www.mdpi.com/article/10.3390/jcm11195940/s1, Table S1: Self-constructed items on social activities (German).

## Abbreviations

CAIDE	Cardiovascular Risk Factors, Ageing and Incidence of Dementia
LSNS-6	Lubben Social Network Scale
GDS	Geriatric Depression Scale
GP	General Practitioner
GPP	GP practice
MoCA	Montreal Cognitive Assessment
SD	Standard Deviation
SNI	Social Network Index
RCT	Randomized Controlled Trial
