# Supplementary material for: Social Network and Participation in Elderly Primary Care Patients in Germany and Associations with Depressive Symptoms—A Cross-Sectional Analysis from the AgeWell.de Study
Source: J Clin Med. 2022 Oct 8;11(19):5940. doi: 10.3390/jcm11195940 (PMC9572848; doi:10.3390/jcm11195940)
Supplement: Supplementary file 1 [file jcm-11-05940-s001.zip › jcm-1934197-supplementary.pdf]

Table S1. Self-constructed items on social activities (German). Corresponding English translation upon request.

Fragen zu Zugehörigkeit zu sozialen Gruppen / Teilnahme an sozialen Aktivitäten

	ja	nein
Haben Sie Hobbies oder besondere Interessen, bei denen Sie mit anderen Menschen in Kontakt kommen und denen Sie regelmäßig nachgehen (z.B. Karten spielen, Gymnastik, Tanz etc.)?	□	□
Engagieren Sie sich regelmäßig in einer kirchlichen Einrichtung?	□	□
Engagieren Sie sich regelmäßig in einem Verein/Club, einer Partei o. ä.?	□	□
Gehen Sie regelmäßig sozialen Aktivitäten wie z.B. Kino-, Theater-, Restaurant- oder Kneipenbesuch nach?	□	□
Unternehmen Sie hin und wieder Reisen zusammen mit anderen (Familie, Freunde oder organisierte Gruppenreisen)?	□	□
Unternehmen Sie hin und wieder Wander- oder Fahrradtouren zusammen mit anderen?	□	□
Gehen Sie einer ehrenamtlichen Tätigkeit nach?	□	□
Nutzen Sie das Angebot der örtlichen Volkshochschulen?	□	□
Sonstige soziale Aktivitäten (bitte spezifizieren): ______________________________	□	□

**Nehmen Sie regelmäßig (1 Mal pro Woche) an sozialen Aktivitäten teil (z.B. Vereine, Seniorennachmittage, Kirche, Kurse, organisierte Ausflüge, kulturelle Angebote)?**	
□ **Nein,**	□ und ich habe es auch nicht vor.<br>□ aber ich denke darüber nach.<br>□ aber ich habe die feste Absicht dazu.
□ **Ja,**	□ aber es fällt mir schwer.<br>□ und es fällt mir leicht.
